# Editorial: Fibrosis: etiology, pathophysiology, measurements, and therapy

**DOI:** 10.3389/fphar.2023.1201830

**Published:** 2023-05-15

**Authors:** Yanping Liu, Jiupan Xu, Yuxia Zhao, Panpan Hao

**Affiliations:** ^1^ Department of Radiology, Qilu Hospital of Shandong University, Jinan, Shandong, China; ^2^ National Key Laboratory for Innovation and Transformation of Luobing Theory, The Key Laboratory of Cardiovascular Remodeling and Function Research, Chinese Ministry of Education, Chinese National Health Commission and Chinese Academy of Medical Sciences, Department of Cardiology, Qilu Hospital of Shandong University, Jinan, Shandong, China; ^3^ Department of Traditional Chinese Medicine, Qilu Hospital of Shandong University, Jinan, Shandong, China

**Keywords:** fibrosis, ADAM17, melatonin, emodin, salusin-β, hypoxia-inducible factor (HIF)

Various stress injuries can cause organ fibrosis, depending on different pathogeneses and mechanisms. Excessive fibrosis aggravates pathological remodeling and functional deterioration of kidney, heart, lung, liver, etc. Progress has been made in the diagnosis and treatment of fibrosis; for example, the N-terminal propeptide of collagen type I and the amino-terminal peptide of procollagen type III have been shown to correlate with inflammation and fibrosis in the liver but not specifically with the liver (Lurie et al., 2015). In the absence of specific therapeutic targets, circulating biomarkers, and imaging techniques, fibrosis research is challenging.

For this Research Topic, we collected 7 review articles, 8 original research papers, and 2 meta-analyses in the field of organ fibrosis. One review (Yang et al.) focused on endogenous exosomes, as small extracellular vesicles, in the development, evaluation, and treatment of pulmonary fibrosis. Endogenous exosomes are involved in intercellular communication by delivering cellular cargoes to recipient cells, including nucleic acids, functional proteins, metabolites, etc., and in numerous biological processes, such as inflammation, extracellular matrix deposition, and epithelial-mesenchymal transition, showing promise for the evaluation and treatment of pulmonary fibrosis. Another review (Tepus et al.) examined the possibility of using extracellular vesicles to evaluate renal fibrosis. Molecular profiling data showed that extracellular vesicles have potential diagnostic and therapeutic value in chronic kidney disease. Techniques for the application of extracellular vesicles are not yet mature, as there are still obstacles in obtaining, preserving, and loading extracellular vesicles. Simplified and reproducible methods need to be explored for clinical application of extracellular vesicles, especially exosomes. Li et al. summarized novel mechanisms and therapeutic targets for metabolic reprogramming in myofibroblasts, endothelial cells, alveolar epithelial cells and macrophages during pulmonary fibrosis. How the tumor suppressor p53 is involved in the development and progression of liver fibrosis was discussed by Yu et al. Targeted regulation of p53 may be effective in the treatment of liver fibrosis. According to a review article (Liu et al.), cGAS/STING helps the body fight harmful factors, such as microorganisms and cancer, while many reports show the flip side that cGAS/STING enhances immunopathological responses and injury. cGAS/STING overactivation exacerbates immunopathological damage and fibrosis, contributing to a new potential target for fibrosis assessment and intervention. Ding et al. reviewed the effects of bariatric surgery on cardiac, hepatic and renal comorbidities in patients with type 2 diabetes mellitus and the possible mechanisms. Clinical evidence shows that bariatric surgery is superior to drug therapy in terms of glycemic control and organ fibrosis, and that bariatric surgery has long-term benefits for cardiac, hepatic, renal, and other organ function by reversing fibrosis. Thus, bariatric surgery is recommended for the treatment of severely obese patients with type 2 diabetes. Yin et al. discussed the pathophysiology, evaluation, and intervention of postinfarction myocardial fibrosis. Regional fibrosis in infarcted zones and collagen deposition in noninfarcted zones promote pathological ventricular remodeling. Reperfusion in combination with new drugs such as angiotensin receptor neprilysin inhibitors and sodium/glucose cotransporter 2 inhibitors, as well as mineralocorticoid receptor antagonists and β-adrenoceptor antagonists, counteracts pathological ventricular remodeling and delays heart failure. Promising therapeutic options such as anti-inflammatory agents, bone marrow-derived cells, and microRNAs are being explored.

**FIGURE 1:**
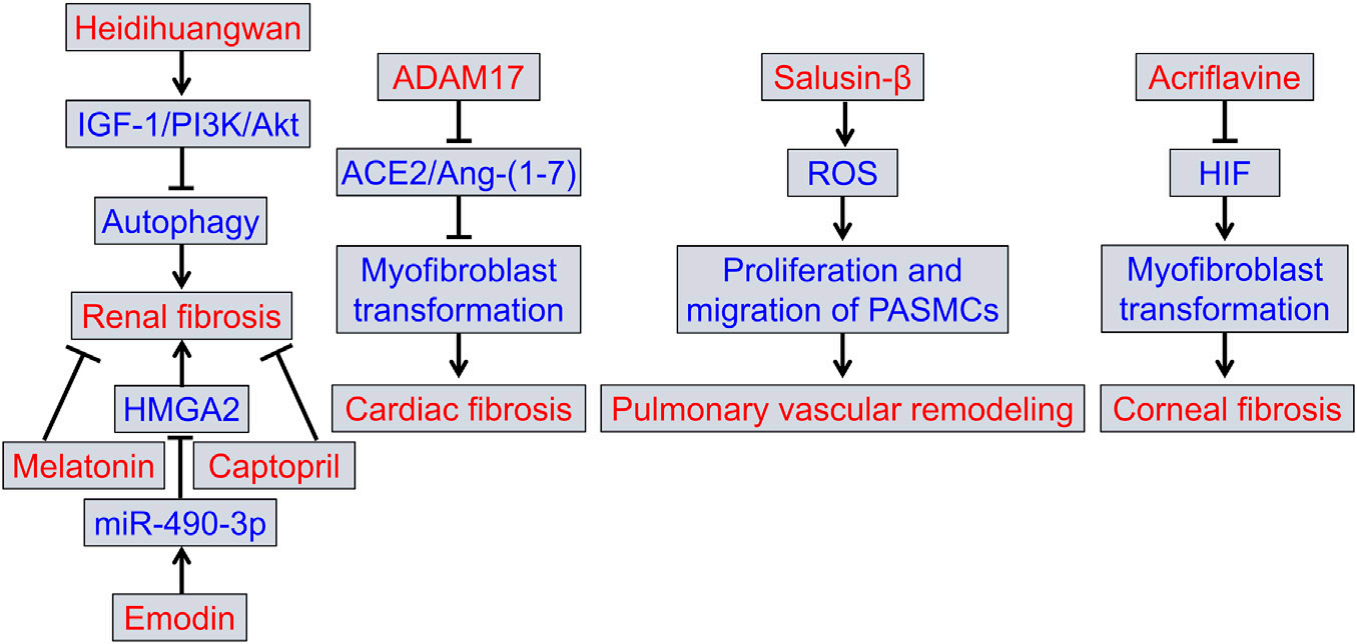
Summary of the preclinical studies in this Research Topic. ACE2, angiotensin-converting enzyme 2; ADAM17, a disintegrin and metalloprotease domain family protein 17; Ang-(1–7), angiotensin-(1–7); HIF, hypoxia-inducible factor; HMGA2, high mobility protein A2; IGF-1, insulin-like growth factor 1; PASMCs, pulmonary arterial smooth muscle cells; ROS, reactive oxygen species.

Six preclinical studies addressed the mechanisms and therapeutic strategies for organ fibrosis (Figure 1). Among them, three studies investigated therapeutic strategies for renal fibrosis (Li et al.; Repova et al.; Wang et al.). One herbal formulation, Heidihuangwan, was shown to improve renal fibrosis and functional deterioration in rats with 5/6 nephrectomy (Li et al.). Heidihuangwan inhibited autophagy through increasing insulin-like growth factor 1 level and its binding to its specific receptor and activating the PI3K/Akt signaling pathway, resulting in attenuation of renal fibrosis. Another study investigated whether lactacystin induces fibrotic renal remodeling and how melatonin and captopril affect these changes (Repova et al.). Lactacystin damaged rat kidneys, and treatment with melatonin or captopril increased glomerular density, decreased glomerular tuft area, and decreased hydroxyproline levels in kidneys. Melatonin or captopril therapy decreased intraglomerular and tubulointerstitial collagen I and III. Because melatonin did not lower blood pressure, the antifibrotic effect was probably achieved by its direct and pleiotropic actions. Considering the results on cardiac fibrosis from other studies and renal fibrosis from this experiment, the model of cardiorenal injury and fibrosis established using lactacystin is promising. In the third study (Wang et al.), investigators examined the role of emodin in renal fibrosis and the underlying mechanisms using a mouse model with unilateral ureteral obstruction and a rat renal tubule epithelial cell line stimulated with TGF-β1. Emodin increased miR-490-3p level, decreased high migration protein A2 level, and attenuated TGF-β1-dependent fibrosis.

With regard to cardiac fibrosis, Cheng et al. found that knockdown of ADAM17 attenuated left ventricular fibrosis and functional deterioration in diabetic mice, whereas overexpression of ADAM17 exacerbated it by shearing angiotensin-converting enzyme 2, downregulating angiotensin-(1–7) production and promoting myofibroblast transformation via the TGF-β1/Smad3 pathway. Targeted regulation of ADAM17/angiotensin-converting enzyme 2/angiotensin-(1–7) may be a promising approach to prevent and treat pathological ventricular remodeling.

A study by Wang et al. confirmed the detrimental effect of salusin-β on monocrotaline-induced pulmonary vascular remodeling. Increased salusin-β activity in pulmonary arteries of rats with pulmonary hypertension contributes to pulmonary artery smooth muscle cell proliferation and migration and vascular remodeling through upregulation of reactive oxygen species.

In a study by Zhu et al., a mouse model with mechanical corneal injury was used to investigate the effects of acriflavine, an inhibitor of hypoxia-inducible factor (HIF) dimerization, on corneal fibrosis and transparency. The results showed that inhibiting HIF alleviated corneal fibrosis and transparency in mechanically injured corneal stroma. Acriflavine inhibited corneal fibrosis by downregulating the TGF-β1 signaling pathway and myofibroblast transformation. In addition, inhibiting HIF also downregulated fibronectin level, which did not depend on the TGF-β1 pathway.

A preclinical meta-analysis (Wu et al.) evaluated the effects of metformin on pulmonary inflammation and fibrosis score in animal models of pulmonary fibrosis. The results showed that metformin can ameliorate pulmonary fibrosis in animals by activating AMPK, reducing TGF-β levels and phosphorylation of Smad2/3 and ERK1/2, and inhibiting fibroblast proliferation and differentiation, epithelial-mesenchymal transition, and inflammation.

This Research Topic included two retrospective clinical studies, one of which examined the relationship between myocardial strain and infarct size in patients with preserved systolic function after myocardial infarction (Wang et al.). The global circumferential strain determined by cardiac magnetic resonance imaging had higher diagnostic accuracy than the global longitudinal strain in assessing infarct size. Park et al. analyzed magnetic resonance elastography of 6215 subjects at average risk for liver fibrosis. The diagnostic value of fibrosis-4 was confirmed to be better than the fibrosis score for nonalcoholic fatty liver disease in screening for liver fibrosis in such average-risk populations.

The efficacy of cordyceps in patients with cardiac arrhythmias and the identification of possible mechanisms have been studied using meta-analysis and network pharmacology (Wang et al.). Cordyceps improves sinus node and atrioventricular conduction by regulating PI3K/Akt and adrenergic signalling pathways in patients with cardiac arrhythmias, with no apparent side effects.

In conclusion, the broad coverage of this Research Topic opens numerous new perspectives on molecular mechanisms, evaluation, and therapeutic approaches in organ fibrosis.
